# Supplementary material for: A potential cephalopod from the early Cambrian of eastern Newfoundland, Canada
Source: Commun Biol. 2021 Mar 23;4:388. doi: 10.1038/s42003-021-01885-w (PMC7987959; doi:10.1038/s42003-021-01885-w)
Supplement: Supplementary file 2 — Supplementary Information [file 42003_2021_1885_MOESM2_ESM.pdf]

Supplementary information for:

A potential cephalopod from the early Cambrian of eastern Newfoundland, Canada

Anne Hildenbrand*[1], Gregor Austermann*[1], Dirk Fuchs[2], Peter Bengtson[1] & Wolfgang Stinnesbeck[1]

[1]Institute of Earth Sciences, Heidelberg University, Im Neuenheimer Feld 234–236, 69120 Heidelberg, Germany
[2]Bavarian State Collection for Paleontology and Geology, Richard-Wagner-Straße 10, 80333 Munich, Germany

*corresponding authors

Supplementary Figure 1: Elements of Small Shelly Fossil fauna accompanying our material in the Bonavista Formation.

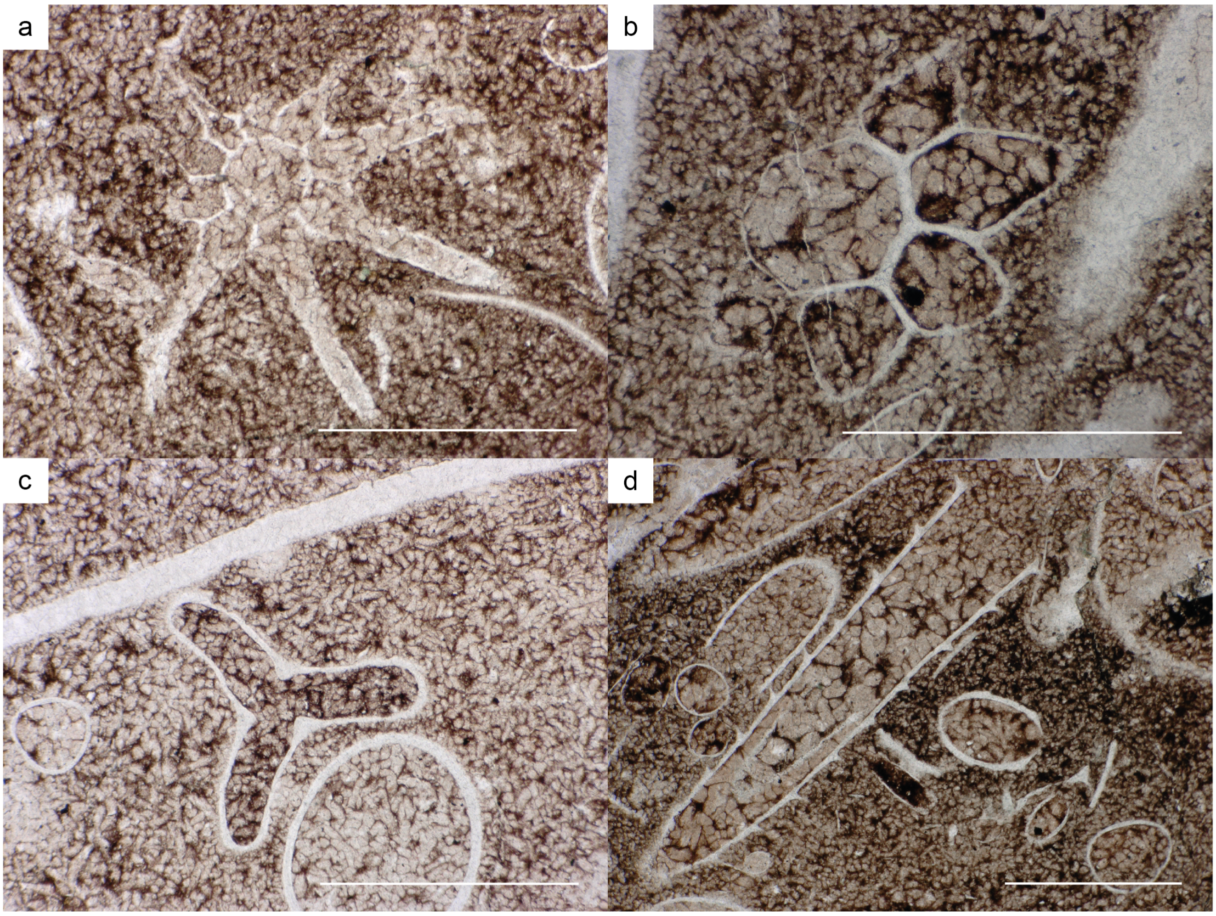

a, **b** Chanceloriids. **c** *Anabarites* sp. **d** Ornamented orthoconic fossil. Scale bar is 1 mm.

Supplementary Figure 2: Colour-coded SEM-EDX element mappings of NFM F-2774.

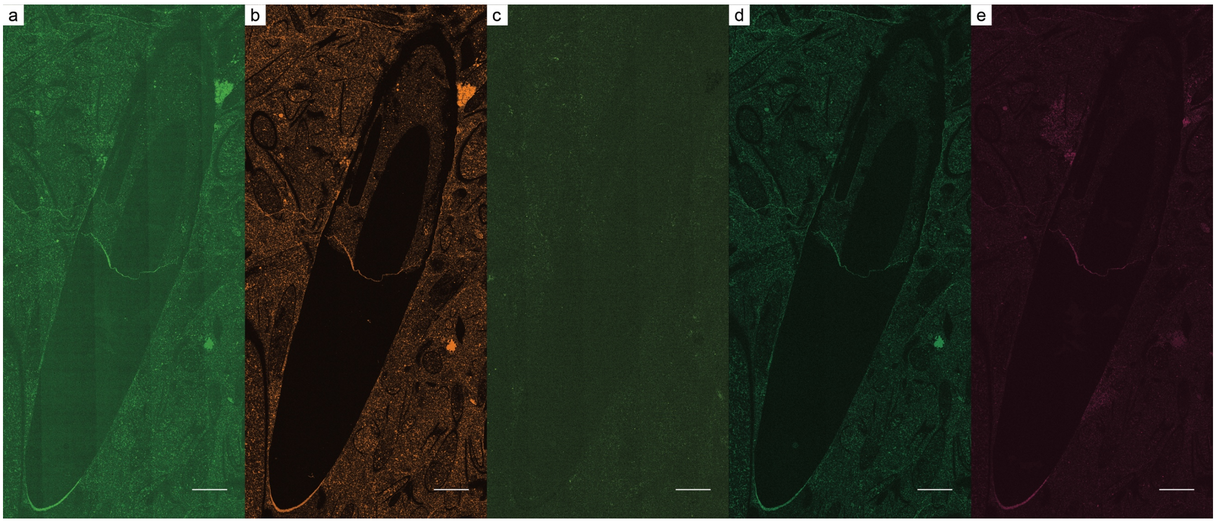

Bright colour indicates high amounts of the respective element. **a** Oxygen. **b** Silicon. **c** Phosphorus. **d** Potassium. **e** Iron. Scale bar is 1 mm.

Supplementary Figure 3: Colour-coded SEM-EDX element mappings of NFM F-2776.

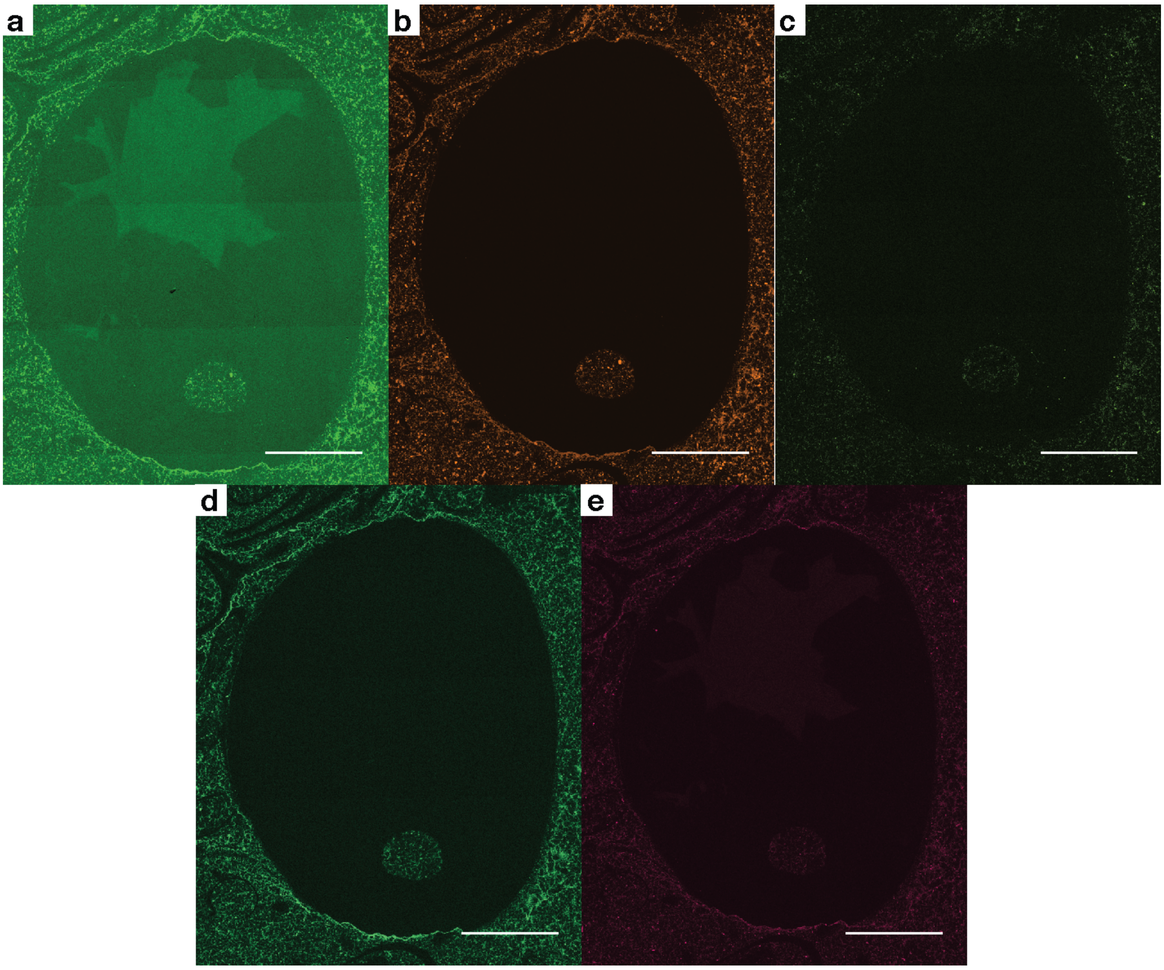

Bright colour indicates high amounts of the respective element. **a** Oxygen. **b** Silicon. **c** Phosphorus. **d** Potassium. **e** Iron. Scale bar is 1 mm.

Supplementary Figure 4: Measurements of our specimens.

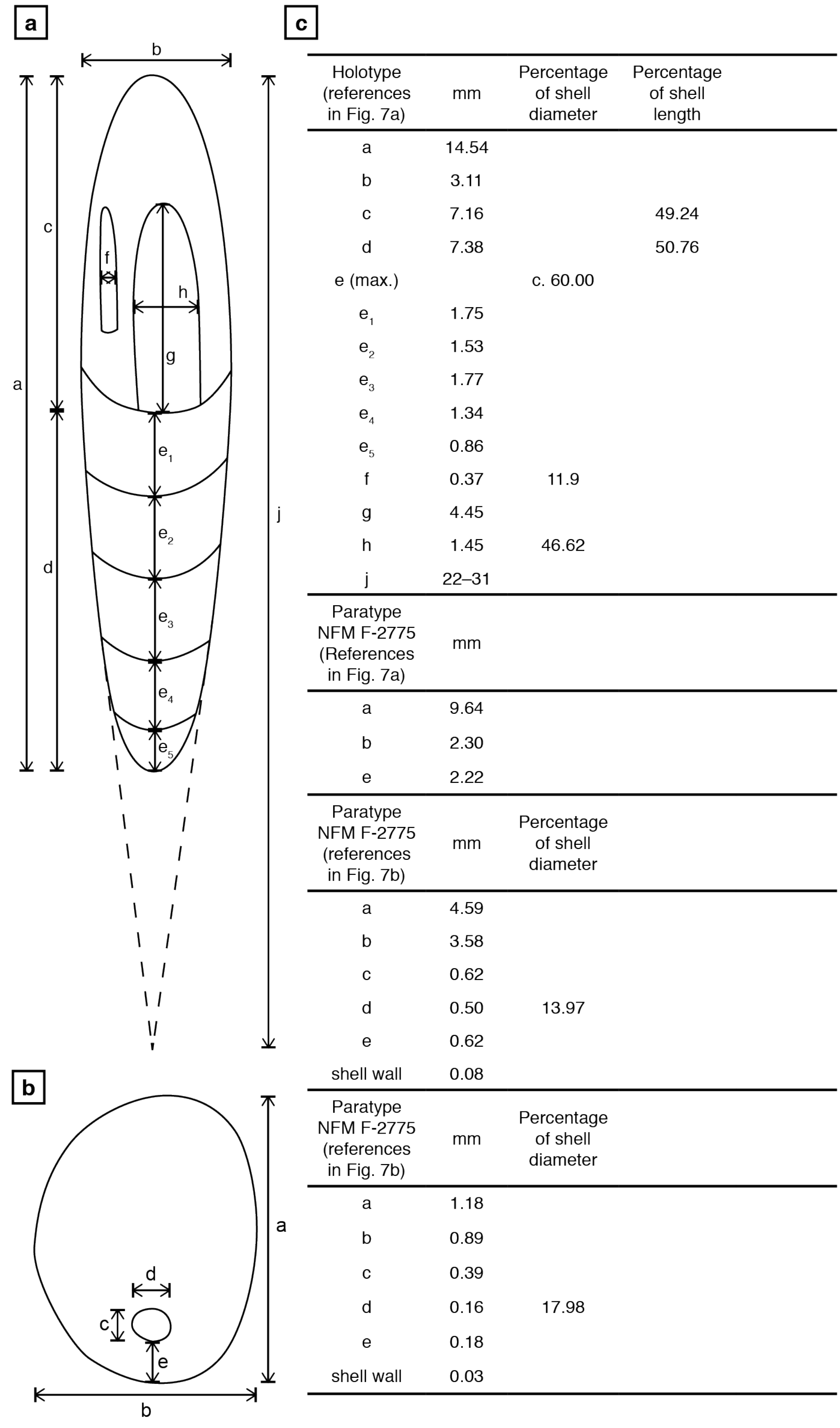

c

Holotype (references in Fig. 7a)	mm	Percentage of shell diameter	Percentage of shell length
a	14.54		
b	3.11		
c	7.16		49.24
d	7.38		50.76
e (max.)		c. 60.00	
e_1	1.75		
e_2	1.53		
e_3	1.77		
e_4	1.34		
e_5	0.86		
f	0.37	11.9	
g	4.45		
h	1.45	46.62	
j	22–31		

Paratype NFM F-2775 (References in Fig. 7a)	mm
a	9.64
b	2.30
e	2.22

Paratype NFM F-2775 (references in Fig. 7b)	mm	Percentage of shell diameter
a	4.59	
b	3.58	
c	0.62	
d	0.50	13.97
e	0.62	
shell wall	0.08	

Paratype NFM F-2775 (references in Fig. 7b)	mm	Percentage of shell diameter
a	1.18	
b	0.89	
c	0.39	
d	0.16	17.98
e	0.18	
shell wall	0.03	

a NFM F-2774 and NFM F-2775 and F-2776. **b** NFM F-2777. Figure not to scale. **c** Table showing measured values in the material described here.
