# Supplementary material for: A potential cephalopod from the early Cambrian of eastern Newfoundland, Canada
Source: Commun Biol. 2021 Mar 23;4:388. doi: 10.1038/s42003-021-01885-w (PMC7987959; doi:10.1038/s42003-021-01885-w)
Supplement: Supplementary file 3 — Reporting Summary [file 42003_2021_1885_MOESM3_ESM.pdf]

nature research

Corresponding author(s): Anne Hildenbrand (anne.hildenbrand@geow.uni-heidelberg.de) Gregor Austermann (gregor.austermann@geow.uni-heidelberg.de)

Last updated by author(s): Feb 13, 2021

Reporting Summary

Nature Research wishes to improve the reproducibility of the work that we publish. This form provides structure for consistency and transparency in reporting. For further information on Nature Research policies, see our Editorial Policies and the Editorial Policy Checklist.

Statistics

For all statistical analyses, confirm that the following items are present in the figure legend, table legend, main text, or Methods section.

n/a	Confirmed	
☒	☐	The exact sample size (*n*) for each experimental group/condition, given as a discrete number and unit of measurement
☒	☐	A statement on whether measurements were taken from distinct samples or whether the same sample was measured repeatedly
☒	☐	The statistical test(s) used AND whether they are one- or two-sided<br>*Only common tests should be described solely by name; describe more complex techniques in the Methods section.*
☒	☐	A description of all covariates tested
☒	☐	A description of any assumptions or corrections, such as tests of normality and adjustment for multiple comparisons
☒	☐	A full description of the statistical parameters including central tendency (e.g. means) or other basic estimates (e.g. regression coefficient) AND variation (e.g. standard deviation) or associated estimates of uncertainty (e.g. confidence intervals)
☒	☐	For null hypothesis testing, the test statistic (e.g. *F*, *t*, *r*) with confidence intervals, effect sizes, degrees of freedom and *P* value noted<br>*Give P values as exact values whenever suitable.*
☒	☐	For Bayesian analysis, information on the choice of priors and Markov chain Monte Carlo settings
☒	☐	For hierarchical and complex designs, identification of the appropriate level for tests and full reporting of outcomes
☒	☐	Estimates of effect sizes (e.g. Cohen's *d*, Pearson's *r*), indicating how they were calculated

Our web collection on statistics for biologists contains articles on many of the points above.

Software and code

Policy information about availability of computer code

Data collection	SEM-EDS data was collected using AZTEC 4.2. No custom algorithms were used in this study.
Data analysis	SEM-EDS data was analysed using AZTEC 4.2. No custom algorithms were used in this study.

For manuscripts utilizing custom algorithms or software that are central to the research but not yet described in published literature, software must be made available to editors and reviewers. We strongly encourage code deposition in a community repository (e.g. GitHub). See the Nature Research guidelines for submitting code & software for further information.

Data

Policy information about availability of data

All manuscripts must include a data availability statement. This statement should provide the following information, where applicable:

- Accession codes, unique identifiers, or web links for publicly available datasets
- A list of figures that have associated raw data
- A description of any restrictions on data availability

All specimens are housed in the Provincial Museum Division, The Rooms Corporation of Newfoundland and Labrador, St. John's, Newfoundland, Canada (NFM). The authors declare that the data supporting the findings of this study are available within the article and its supplementary information files.

Field-specific reporting

Please select the one below that is the best fit for your research. If you are not sure, read the appropriate sections before making your selection.

☐ Life sciences ☐ Behavioural & social sciences ☒ Ecological, evolutionary & environmental sciences

For a reference copy of the document with all sections, see nature.com/documents/nr-reporting-summary-flat.pdf

Ecological, evolutionary & environmental sciences study design

All studies must disclose on these points even when the disclosure is negative.

Study description	We present new material from the early Cambrian of Newfoundland that potentially backdates the evolution of cephalopods to before euarthropods. The material is most closely aligned with the Cephalopoda, lending a tentative assignment and significant potential implications for the origin of this molluscan group. We used optical microscopy and energy dispersive spectroscopy to analyse four specimens hosted in limestone from a siliciclastic succession at Bacon Cove, eastern Newfoundland, Canada.
Research sample	Samples were newly collected from the rocks at Bacon Cove and prepared at Heidelberg University, Heidelberg, Germany. All specimens are preserved as thin sections and tentatively assigned to the Cephalopoda. The following accession numbers were assigned to the material: NFM F-2774 – NFM F-2777.
Sampling strategy	Samples were in part visible in the outcrop. Thus, sampling was undertaken at these spots.
Data collection	The study is based on field work carried out by AH and GA. Field documentation was done by AH and GA and is reported in Fig. 5.
Timing and spatial scale	June, 1st to July, 31st 2016, Bacon Cove, eastern Newfoundland
Data exclusions	No data was excluded.
Reproducibility	Tentative assignment possible in four specimens. Reproducibility failed in the remaining material.
Randomization	Randomization is not relevant here, as no statistical analyses were done.
Blinding	Blinding is not relevant here, as no statistical analyses were done.

Did the study involve field work? ☒ Yes ☐ No

Field work, collection and transport

Field conditions	Samples were cut out from fresh rock. Environmental conditions further than diagenesis do not apply to samples.
Location	The fossil site at Bacon Cove is situated in a small cove (Lower Cove) on the southwestern side of Conception Bay (47°29'05.3"N 53°09'58.1"W), eastern Newfoundland, Canada, on lands belonging to the Canadian Crown. The outcrop spans c. 200 m of the NNW–SSE-aligned coast.
Access & import/export	The samples were transported to Germany. No restriction prohibited the transport, permissions were not required.
Disturbance	No disturbance was recorded.

Reporting for specific materials, systems and methods

We require information from authors about some types of materials, experimental systems and methods used in many studies. Here, indicate whether each material, system or method listed is relevant to your study. If you are not sure if a list item applies to your research, read the appropriate section before selecting a response.

Materials & experimental systems

n/a	Involved in the study	
☒	☐	Antibodies
☒	☐	Eukaryotic cell lines
☐	☒	Palaeontology and archaeology
☒	☐	Animals and other organisms
☒	☐	Human research participants
☒	☐	Clinical data
☒	☐	Dual use research of concern

Methods

n/a	Involved in the study	
☒	☐	ChIP-seq
☒	☐	Flow cytometry
☒	☐	MRI-based neuroimaging

Palaeontology and Archaeology

Specimen provenance	The fossil site at Bacon Cove is situated in a small cove (Lower Cove) on the southwestern side of Conception Bay (47°29'05.3"N 53° 09'58.1"W), eastern Newfoundland, Canada, on lands belonging to the Canadian Crown. The outcrop spans c. 200 m of the NNW–SSE-aligned coast. No permit was required for the field work.
Specimen deposition	All specimens are housed in the Provincial Museum Division, The Rooms Corporation of Newfoundland and Labrador, St. John's, Newfoundland, Canada (NFM). Specimen accession numbers: NFM F-2774 – NFM F-2777.
Dating methods	No new dates are provided.

☒ Tick this box to confirm that the raw and calibrated dates are available in the paper or in Supplementary Information.

Ethics oversight	No ethical approval or guidance was required, as no animals humans or any living beeings were included in this study.

Note that full information on the approval of the study protocol must also be provided in the manuscript.
